# Insulin Sensitivity and Associated Plasma Proteomics During Sex Hormone Therapy

**DOI:** 10.1210/clinem/dgaf573

**Published:** 2025-10-22

**Authors:** Sarah A van Eeghen, Laura Pyle, Phoom Narongkiatikhun, Ye Ji Choi, Taryn G Vosters, Irene G M van Valkengoed, Petter Bjornstad, Sarah E Siegelaar, Natalie J Nokoff, Martin den Heijer, Daniël H van Raalte

**Affiliations:** Center of Expertise on Gender Dysphoria, Department of Internal Medicine, Amsterdam UMC, location Vrije Universiteit, 1081 HV Amsterdam, The Netherlands; Amsterdam Gastroenterology Endocrinology Metabolism, Amsterdam UMC, location University of Amsterdam, 1105 AZ Amsterdam, The Netherlands; Department of Endocrinology and Metabolism, Amsterdam UMC, location Vrije Universiteit Amsterdam, 1081 HV Amsterdam, The Netherlands; Department of Medicine, Division of Endocrinology, Metabolism and Nutrition, University of Washington School of Medicine, Seattle, WA 98195, USA; Department of Pediatrics, Section of Endocrinology, University of Colorado School of Medicine, Aurora, CO 80045, USA; Department of Medicine, Division of Endocrinology, Metabolism and Nutrition, University of Washington School of Medicine, Seattle, WA 98195, USA; Division of Nephrology, Department of Internal Medicine, Faculty of Medicine, Chiang Mai University, Chiang Mai 50200, Thailand; Department of Medicine, Division of Endocrinology, Metabolism and Nutrition, University of Washington School of Medicine, Seattle, WA 98195, USA; Department of Pediatrics, Section of Endocrinology, University of Colorado School of Medicine, Aurora, CO 80045, USA; Department of Public and Occupational Health, Amsterdam UMC, location University of Amsterdam, 1105 AZ Amsterdam, The Netherlands; Department of Public and Occupational Health, Amsterdam UMC, location University of Amsterdam, 1105 AZ Amsterdam, The Netherlands; Department of Medicine, Division of Endocrinology, Metabolism and Nutrition, University of Washington School of Medicine, Seattle, WA 98195, USA; Department of Pediatrics, Division of Endocrinology, University of Washington School of Medicine, Seattle, WA 98105, USA; Amsterdam Gastroenterology Endocrinology Metabolism, Amsterdam UMC, location University of Amsterdam, 1105 AZ Amsterdam, The Netherlands; Department of Endocrinology and Metabolism, Amsterdam UMC, location Vrije Universiteit Amsterdam, 1081 HV Amsterdam, The Netherlands; Department of Pediatrics, Section of Endocrinology, University of Colorado School of Medicine, Aurora, CO 80045, USA; Center of Expertise on Gender Dysphoria, Department of Internal Medicine, Amsterdam UMC, location Vrije Universiteit, 1081 HV Amsterdam, The Netherlands; Amsterdam Gastroenterology Endocrinology Metabolism, Amsterdam UMC, location University of Amsterdam, 1105 AZ Amsterdam, The Netherlands; Department of Endocrinology and Metabolism, Amsterdam UMC, location Vrije Universiteit Amsterdam, 1081 HV Amsterdam, The Netherlands; Department of Endocrinology and Metabolism, Amsterdam UMC, location Vrije Universiteit Amsterdam, 1081 HV Amsterdam, The Netherlands; Diabetes Center, Amsterdam UMC, location Vrije Universiteit, 1081 HV Amsterdam, The Netherlands; Amsterdam Cardiovascular Sciences, location Vrije Universiteit Amsterdam, 1081 HV Amsterdam, The Netherlands

**Keywords:** estradiol, insulin sensitivity, plasma proteomics, sex hormone therapy, sex hormones, testosterone

## Abstract

**Purpose:**

Women are generally protected against insulin resistance and related comorbidities when compared with men, potentially due to the role of sex hormones. While epidemiological and animal studies suggest that sex hormones may impact insulin sensitivity, studies in humans on the effects of estradiol and testosterone treatment on insulin sensitivity assessed by gold-standard measures remain limited. The molecular mechanisms involved are also not well understood. Therefore, we aimed to investigate changes in insulin sensitivity and associated change in plasma proteome following 3 months of sex hormone therapy.

**Methods:**

This prospective, observational study included 29 individuals initiating feminizing (estradiol with GnRH analogue; n = 16) or masculinizing (testosterone; n = 13) therapy. Measurements at baseline and after 3 months included insulin sensitivity, assessed via hyperinsulinemic-euglycemic clamp (M-value: adjusted for lean body mass and M/I ratio: M-value adjusted for plasma insulin concentrations), along with plasma proteomics.

**Results:**

During feminizing hormone therapy, insulin sensitivity increased (M-value: + 23.3%, M/I ratio: + 20.2%; *P* < .05), whereas during masculinizing hormone therapy, no changes were observed. Of the differentially expressed plasma proteins (49 during feminizing and 356 during masculinizing hormone therapy), 16 correlated with changes in insulin sensitivity. Several were involved in immunoregulation and inflammation (vascular endothelial growth factor D, 5′-nucleotidase), iron homeostasis and erythropoiesis (hepcidin, transferrin receptor protein 1:cytoplasmic domain), and oxidative stress (superoxide dismutase 3).

**Conclusion:**

Feminizing hormone therapy, characterized by high serum estradiol and low serum testosterone concentrations, enhanced insulin sensitivity. These findings highlight the impact of sex hormones on insulin sensitivity and may inform sex-specific precision medicine.

Insulin resistance, defined by impaired responsiveness of the liver, skeletal muscle, and adipose tissue to the metabolic actions of circulating insulin, is a key pathophysiological defect in type 2 diabetes and contributes to other diseases, including cardiovascular and chronic kidney disease (1). Biological sex influences insulin sensitivity and the risk of these conditions, with women generally being more protected compared with men (2). This effect seems largely mediated by the female sex hormone estradiol, as insulin sensitivity decreases after menopause and may be restored through estrogen replacement therapy (3-5). Beyond estradiol, the male sex hormone testosterone also influences insulin sensitivity. In men, lower serum testosterone concentrations are associated with decreased insulin sensitivity, while testosterone therapy may improve insulin sensitivity (6), potentially via aromatization to estradiol (7). Conversely, in postmenopausal women, elevated testosterone concentrations and testosterone therapy have been linked to decreased insulin sensitivity (8, 9). Despite these insights, most research on sex hormones and insulin sensitivity has focused primarily on hypogonadal men or postmenopausal women undergoing hormone replacement therapy, leaving the broader effects of sex hormones in normogonadal individuals understudied.

Transgender individuals receiving sex hormone therapy provide a unique opportunity to study the metabolic effects of controlled changes in sex hormone concentrations. However, prior studies in this population often relied on surrogate measures of insulin sensitivity, such as the homeostatic model assessment of insulin resistance (HOMA-IR) (10-12), which is less suitable for small trials and primarily reflects hepatic rather than whole-body insulin sensitivity (13). Moreover, many studies included cyproterone acetate (CPA) in feminizing hormone therapy regimens (10-12), a drug recently shown to increase HOMA-IR when compared with GnRH analogues (14). This suggests a direct effect of CPA on insulin sensitivity, potentially via glucocorticoid receptor activation (15). The use of CPA as an antiandrogen may therefore have confounded earlier findings on the effects of feminizing hormone therapy on insulin sensitivity. Additionally, the molecular mechanisms through which sex hormones affect insulin sensitivity remain poorly understood (16).

This study addressed these gaps by measuring insulin sensitivity in transgender individuals before and after 3 months of sex hormone therapy, using the gold-standard hyperinsulinemic-euglycemic clamp technique (17). By integrating clinical measurements with plasma proteomics, this study aimed to uncover underlying molecular pathways and gain deeper insight into the metabolic effects of sex hormones.

## Research Design and Methods

The Kidney Function in People Receiving Gender-affirming Hormone Therapy (KNIGHT) study was a prospective, observational study, conducted from April 2021 to June 2023 at the Amsterdam University Medical Center (UMC; The Netherlands) and the University of Colorado Anschutz Medical Campus (CU-AMC; United States). The study was registered with the Dutch Trial Register (ID: NL9517) and ClinicalTrials.gov (ID: NCT04482920). Ethical approval was obtained from the review boards of both institutions, and the study was conducted in accordance with the Declaration of Helsinki and Good Clinical Practice guidelines. All participants provided written informed consent before enrollment.

### Participant Recruitment and Eligibility

**Figure 1.**
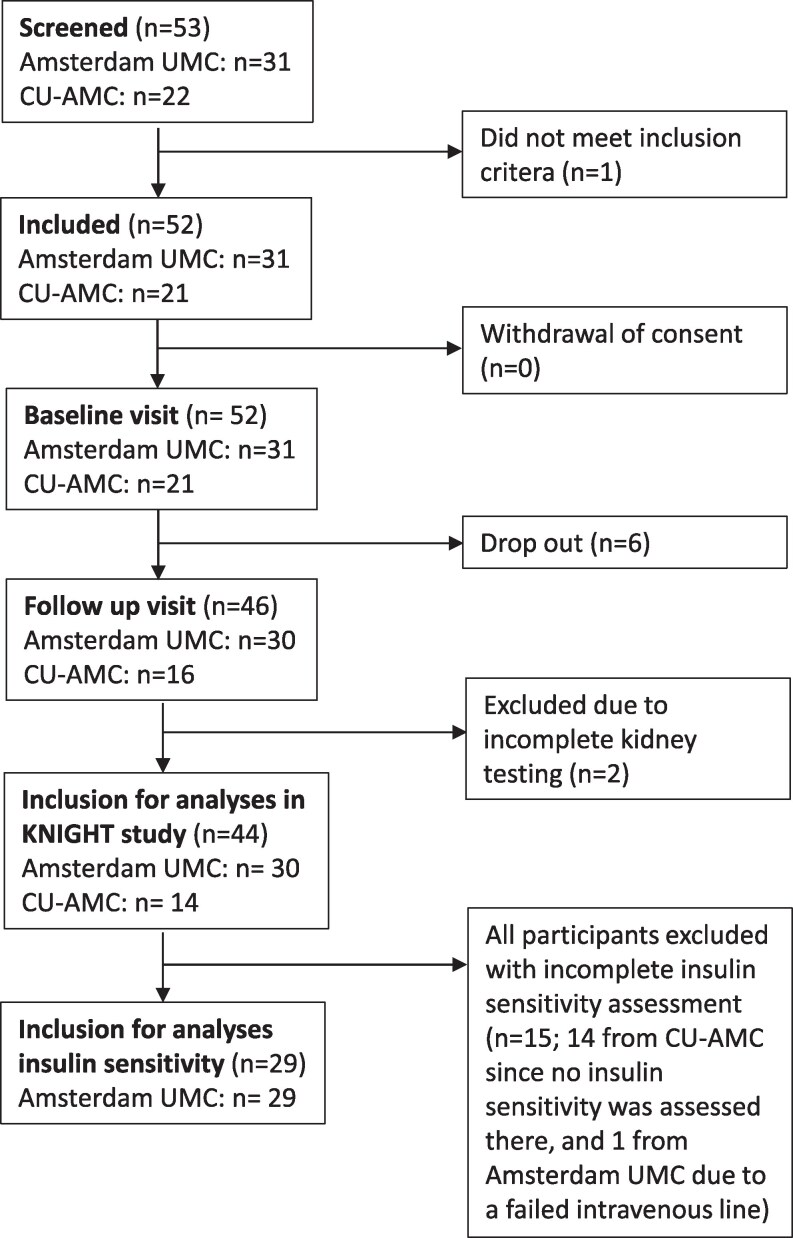
Flowchart of inclusion process. Of the 44 individuals included in the KNIGHT study, plasma proteomics were measured in all participants. Insulin sensitivity analyses were available for 29 individuals (from Amsterdam UMC only, due to logistical reasons). Abbreviations: CU-AMC, University of Colorado Anschutz Medical Campus; KNIGHT, Kidney Function in People Receiving Gender-affirming Hormone Therapy; UMC, University Medical Center.

Participants were recruited through Amsterdam UMC's center of expertise on gender dysphoria and CU-AMC's clinical programs for transgender healthcare. Eligible participants were aged 17 to 40 years, diagnosed with gender dysphoria according to *Diagnostic and Clinical Manual of Mental Disorders* (fifth edition) criteria (18), and scheduled to clinically start sex hormone therapy within 1 month (no medications were administered as a part of the study). Exclusion criteria encompassed cognitive, psychiatric, or physical impairments affecting study participation; previous use of sex hormones, antiandrogens, or history of gonadectomy; pregnancy; concurrent participation in other studies; use of antihypertensive medication; kidney disease; diabetes; uncontrolled hypertension; cardiovascular disease; or iodine-related allergies. A total of 44 participants completed both study visits, including plasma proteomics measurements. Of these, 23 underwent feminizing hormone therapy (16 at Amsterdam UMC and 7 at CU-AMC) and 21 underwent masculinizing hormone therapy (14 at Amsterdam UMC and 7 at CU-AMC). Insulin sensitivity was only assessed in the Amsterdam UMC due to logistical reasons (n = 29; 16 underwent feminizing hormone therapy and 13 underwent masculinizing hormone therapy (Fig. 1).

### Treatment Protocol

Sex hormone therapy was prescribed and administered according to established local clinical protocols at each study site.

At Amsterdam UMC, feminizing hormone therapy consisted of oral estradiol (4 mg daily) or a transdermal estradiol patch (100 mcg per 24 hours), combined with the GnRH analogue triptorelin (3.75 mg intramuscularly every 4 weeks) as an antiandrogen. Masculinizing hormone therapy included transdermal testosterone gel (40.5 mg once daily) or intramuscular testosterone, comprised of a blend of 30 mg testosterone propionate, 60 mg phenylpropionate, 60 mg isocaproate, and 100 mg decanoate, administered every 3 weeks.

At CU-AMC, feminizing hormone therapy was initiated with oral estradiol (0.5-1 mg daily) or a transdermal estradiol patch (50 mcg per 24 hours), combined with antiandrogen therapy using either spironolactone (100-200 mg daily) or finasteride (1.25 mg once daily). Masculinizing hormone therapy included transdermal testosterone gel (20.25-40.5 mg once daily) or subcutaneous testosterone cypionate (20-30 mg once weekly).

Treatment adherence was monitored during routine clinical visits.

### Study Visits and Procedures

Participants attended 2 study visits: baseline and after 3 months of sex hormone therapy. At least 3 days prior to each visit, participants followed “normal” sodium (9-12 g/day) and protein (1.5-2.0 g/kg/day) diets. They were instructed to avoid vigorous physical activity and alcohol for at least 24 hours and to refrain from caffeine consumption for at least 12 hours.

During each visit, 12-hour fasting morning blood samples were collected. Serum estradiol, testosterone, hemoglobin A1c (only at baseline), and glucose were measured immediately. Remaining plasma was stored at −80 °C and analyzed in batches for proteomics after study completion.

Kidney function tests were performed to measure glomerular filtration rate and effective renal plasma flow. These outcomes have been published in a separate publication (19). During kidney testing, bioimpedance analysis (single-frequency bioelectrical impedance analyzer, Maltron BF-906, Maltron International, Essex, UK) was conducted to assess fat and lean body mass.

After kidney testing, a hyperinsulinemic-euglycemic clamp was initiated (at the Amsterdam UMC site). Insulin (NovoRapid, Novo Nordisk, Denmark) was infused at a rate of 40 mU/min⋅m² for 120 minutes, while plasma glucose was maintained at 5.0 mmol/L via a variable 20% glucose infusion. Blood glucose concentrations were monitored every 5 minutes.

Insulin sensitivity was assessed using the glucose infusion rate during the final 30 minutes of the clamp (steady-state period) and corrected for lean body mass (M-value; mg/kg_lean_/min). Additionally, insulin sensitivity normalized by steady-state insulin (M/I ratio) was calculated by dividing the M-value by the mean insulin concentration during the steady-state period, reflecting the amount of glucose metabolized per unit of plasma insulin.

### Laboratory Measurements

At Amsterdam UMC, total estradiol concentrations were determined using liquid chromatography–tandem mass spectrometry (LC-MS/MS), with an interassay coefficient of variation of 7% and a limit of quantification of 5.45 pg/mL. Total testosterone concentrations were also analyzed via LC-MS/MS, with an interassay coefficient of variation ranging from 4% to 9% and a limit of quantification of 0.1 nmol/L. At CU-AMC, estradiol and testosterone concentrations were similarly assessed using LC-MS/MS at Esoterix LabCorps.

### Plasma Proteomics

Plasma protein concentrations at baseline and at 3-month follow-up were measured using the SOMAscan 7 K Proteomic platform (SomaLogic, Inc.) at Washington University in St. Louis, Missouri, USA. Each sample included internal controls and was normalized to account for both intra- and interplate variation. The SOMAscan 7 K platform utilizes 7604 aptamers to target 6596 human proteins (19).

### Statistical Analysis

#### Sample size determination

The sample size (n = 44) was based on the KNIGHT study's primary objective, which has been reported elsewhere (19).

The current paper reports the secondary endpoints: changes in plasma proteomics, measured in all KNIGHT participants (n = 44), and insulin sensitivity, assessed only in Amsterdam UMC participants (n = 29) during 3 months of sex hormone therapy. As the focus is on insulin sensitivity, clinical outcomes are shown for the 29 participants who completed these assessments, while plasma proteomics results are presented for all participants (n = 44).

#### Baseline characteristics and clinical outcomes

Statistical analyses were performed using STATA® (version 17.0). Baseline characteristics of the study sample (n = 29) were summarized using percentages and frequencies for categorical variables, means (±SD) for normally distributed data and medians [interquartile range (IQR)] for nonnormally distributed variables, assessed through visual inspection of histograms and comparisons of means and medians. For comparison, baseline characteristics and sex hormone concentrations before and during sex hormone therapy for the full KNIGHT cohort (n = 44) are provided as a supplement (20).

To assess changes between baseline and 3 months, individuals undergoing feminizing and masculinizing hormone therapy were analyzed separately. Serum estradiol and testosterone concentrations at both timepoints were compared using the Wilcoxon signed-rank test. Changes in body mass index (BMI), body composition, and insulin sensitivity were analyzed using linear mixed models, with normality evaluated via visual inspection of residual histograms.

Spearman's rank correlations were used to examine associations between changes in sex hormone concentrations and clinical outcomes. Specifically, correlations were assessed between delta (Δ) serum testosterone and estradiol and Δ BMI, fat mass, lean body mass, M-value, and M/I ratio. Additionally, correlations between Δ hematocrit, and Δ M-value and M/I ratio were analyzed.

#### Plasma proteomics

Proteomic data were analyzed using R (version 4.4.0, R Core Team, Vienna). Before analysis, protein measurements were log-transformed and standardized by dividing each value by the SD of that protein within the sample. Changes in proteins between baseline and 3 months were assessed using linear models with moderated *t*-statistics, analyzed separately for individuals undergoing feminizing and masculinizing hormone therapy. *P*-values were adjusted to maintain a false discovery rate of 5% (19).

Exploratory analyses were conducted to identify proteins specifically associated with insulin sensitivity by examining correlations between protein changes and changes in M-value using Spearman's rank correlation. Additionally, Spearman's rank correlations were used to assess relationships between protein changes and Δ serum testosterone and estradiol concentrations. Since these analyses were exploratory, no adjustments for multiple testing were applied.

Pathway analyses were performed using Ingenuity Pathway Analysis (QIAGEN, Hilde).

## Results

Baseline characteristics of the participants included in the insulin sensitivity analysis (n = 29) are presented in Table 1, stratified by feminizing and masculinizing hormone therapy. Baseline characteristics for the entire KNIGHT cohort (n = 44) were comparable [Table S1 (20)].

**Table 1.** Baseline characteristics of the participants included in the KNIGHT study

Variable	Individuals scheduled to start feminizing hormone therapy (n = 16)	Individuals scheduled to start masculinizing hormone therapy (n = 13)
Age, years	27 (22-31)	20 (19-23)
Current smoker, n (%)	3 (19)	5 (38)
BMI, kg/m^2^	27.1 (±7.3)	24.4 (±4.6)
Hematocrit, L/L	0.43 (±0.03)	0.38 (±0.01)
Estradiol, pmol/L	73 (63-91)	162 (125-302)
Testosterone, nmol/L	14.5 (9.2-19.5)	1.0 (0.8-1.1)
Fasting plasma glucose, mmol/L	5.2 (±0.5)	4.9 (±0.5)
HbA1c, %	5.1 (±0.3)	5.1 (±0.2)
HbA1c, mmol/mol	32 (±3)	32 (±2)
Fat mass, kg	30.2 (±13.5)	23.3 (±8.7)
Fat mass, %	32 (±7)	32 (±6)
Lean body mass, kg	59.5 (±13.4)	43.4 (±4.9)
Lean body mass, %	68 (±7)	68 (±6)
M-value, mg/kg_lean_ · min	9.7 (±4.5)	12.4 (±5.4)
M/I ratio, mg/kg_lean_ · min · (pmol/L)^−1^	0.016 (±0.010)	0.019 (±0.009)
Use of medication, n		
Progestogens	0	2
Attention deficit hyperactivity disorder medication	2	3
Antiallergy medication	2	0
Asthma medication	1	0
Antidepressants	3	0
Omeprazole	1	0

Abbreviations: BMI, body mass index; HbA1c, hemoglobin A1c; KNIGHT, Kidney Function in People Receiving Gender-affirming Hormone Therapy; VEGF-D, vascular endothelial growth factor D.

Categorical variables are presented as n (%). Unless otherwise indicated, data are presented according to their distribution, median (interquartile range), and mean (±SD).

After baseline visits, among the individuals included in the insulin sensitivity analysis (n = 29), 15 of the 16 participants initiated feminizing hormone therapy with estradiol via transdermal patches, while 1 received estradiol orally. Additionally, all participants initiating feminizing hormone therapy were prescribed the antiandrogen triptorelin, a GnRH analogue. For participants initiating masculinizing hormone therapy, 12 of the 13 received testosterone via transdermal gel, while 1 received testosterone through intramuscular injections of a testosterone blend.

Details of the feminizing and masculinizing hormone therapies for the entire KNIGHT cohort (n = 44) can be found in Table S2 (20).

### Sex Hormone Concentrations

**Figure 2.**
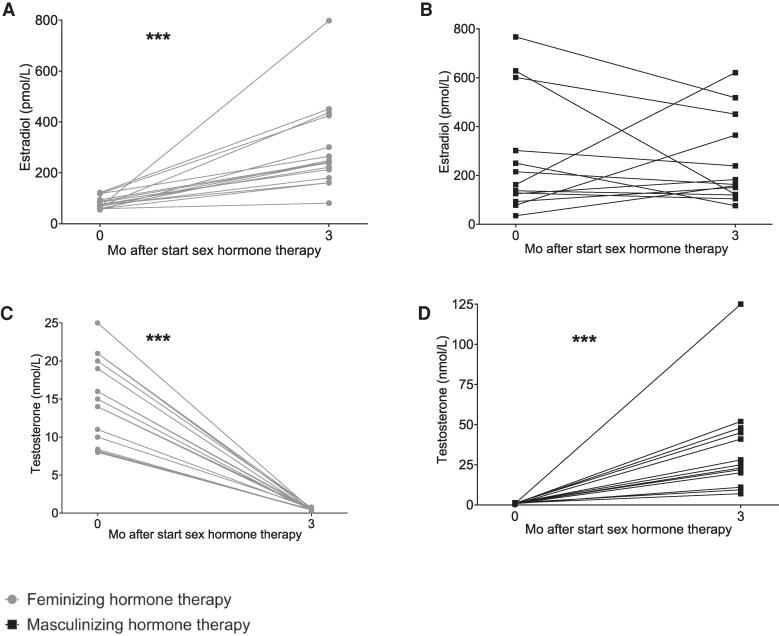
Serum estradiol concentrations (A, B) and serum testosterone concentrations (A, D) before and during 3 months of feminizing (A, C) and masculinizing (B, D) hormone therapy. ***P < .001.

For those undergoing feminizing hormone therapy (n = 16), median (IQR) estradiol concentrations increased from 73 (63-91) to 242 (197-363) pmol/L (*P* < .001), while median (IQR) testosterone concentrations decreased from 14.5 (9.2-19.5) to 0.5 (0.4-0.6) nmol/L (*P* < .001; Fig. 2). For individuals undergoing masculinizing hormone therapy (n = 13), median (IQR) estradiol concentrations at the 3-month follow-up did not significantly differ from baseline [3-month follow up: 163 pmol/L (122-365); *P* = .69], whereas median (IQR) testosterone concentrations increased from 1.0 (0.8-1.1) to 25 (20-45) nmol/L (*P* < .001; Fig. 2). One participant exhibited an unexpectedly high serum testosterone concentration (125 nmol/L) at the 3-month study visit, despite reporting a daily dose of 40.5 mg of transdermal testosterone (Fig. 2). This discrepancy may be attributed to external contamination of the gel (21). As a result, this testosterone measurement was excluded from all subsequent analyses.

Changes in sex hormone concentrations were comparable for the entire KNIGHT cohort [Table S3 (20)].

### Body Composition and Insulin Sensitivity

**Figure 3.**
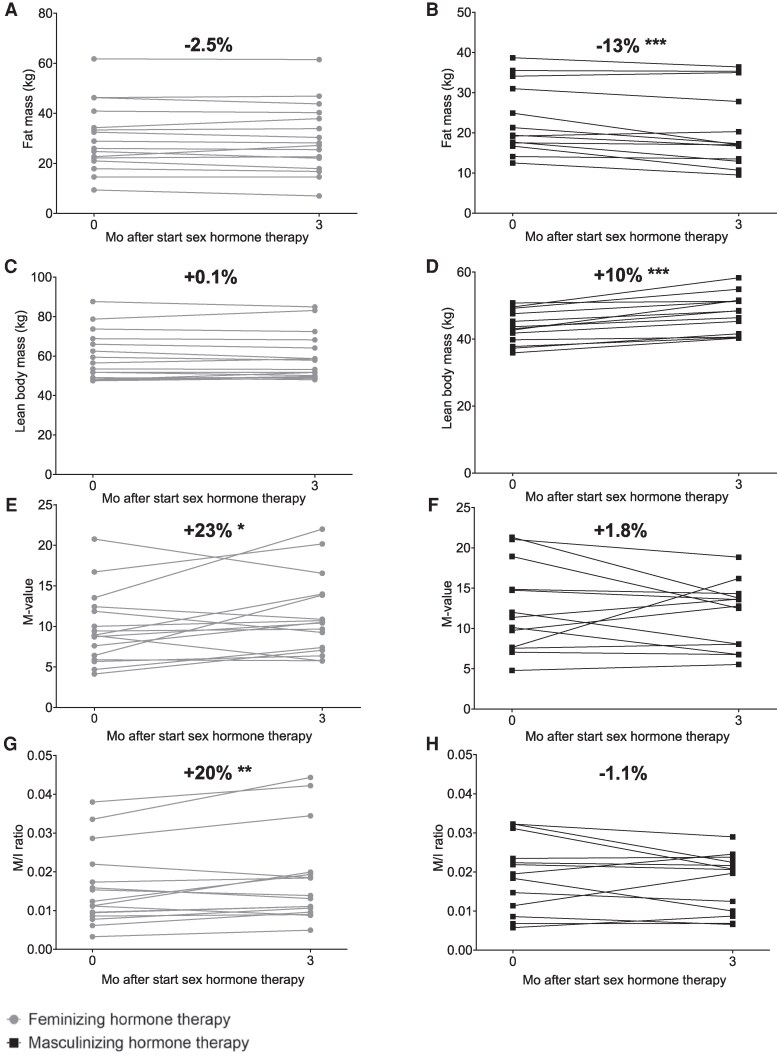
Fat mass (A, B), lean body mass (C, D), M-value (E, F), and M/I ratio (G, H) before and during 3 months of feminizing (A, C, E, G) and masculinizing (B, D, F, H) hormone therapy, with the percentage changes. *P < .05, **P < .01, ***P < .001.

BMI remained unchanged during feminizing hormone therapy, while it increased during masculinizing hormone therapy (Table 2). Among individuals undergoing feminizing hormone therapy, fat mass and lean body mass both remained similar (Table 2, Fig. 3). Conversely, in individuals undergoing masculinizing hormone therapy, fat mass decreased while lean body mass increased (Table 2, Fig. 3).

**Table 2.** Mean (±SD) body composition and insulin sensitivity before and during 3 months of feminizing and masculinizing hormone therapy, with absolute and percentage change

Variables	Feminizing hormone therapy				Masculinizing hormone therapy			
	Baseline	3 mo of HT	Absolute change (95% CI)	% change (95% CI)	Baseline	3 mo of HT	Absolute change (95% CI)	% change (95% CI)
BMI, kg/m^2^	27.1 (±7.3)	27.0 (±7.3)	−0.1 (−0.3 to 0.1)	−0.4 (−1.3 to 0.4)	24.4 (±4.6)	25.0 (±5.0)	0.6 (0.2 to 1.0)	2.3 (0.7 to 3.9)
Fat mass, kg	30.2 (±13.5)	29.8 (±13.9)	−0.4 (−1.4 to 0.6)	−2.5 (−7.3 to 2.3)	23.3 (±8.7)	20.7 (±9.6)	−2.6 (−4.0 to −1.1)	−12.9 (−20.0 to −5.8)
Lean body mass, kg	59.5 (±13.4)	59.4 (±12.0)	−0.1 (−1.1 to 1.0)	0.1 (−1.7 to 1.8)	43.4 (±4.9)	47.6 (±5.8)	4.2 (2.9 to 5.5)	9.7 (6.7 to 12.6)
M-value, mg/kg_lean_ · min	9.7 (±4.5)	11.3 (±4.9)	1.5 (−0.2 to 3.2)	23.3 (3.9 to 42.6)	12.4 (±5.4)	11.6 (±4.1)	−0.8 (−3.0 to 1.4)	1.8 (−18.9 to 22.5)
M/I ratio, mg/kg_lean_ · min · (pmol/L)^−1^	0.0156 (± 0.0100)	0.0181 (±0.0112)	0.0024 (0.0005-0.0044)	20.2 (6.1 to 34.3)	0.0191 (±0.0093)	0.0175 (±0.0075)	−0.0016 (−0.0045 to 0.0012)	−1.1 (−18.8 to 16.5)

Abbreviations: BMI, body mass index; CI, confidence interval; HT, hormone therapy.

Among individuals undergoing feminizing hormone therapy, M-value increased by 23% [95% confidence interval (CI), 4-43; Table 2, Fig. 3], corresponding to a trend toward an absolute increase of 1.5 mg/kg_lean_ · min (95% CI, −0.2-3.2). Similarly, the M/I ratio increased by 20% (95% CI, 6 to 34; Table 2, Fig. 3), corresponding to an absolute increase of 0.0024 mg/kg_lean_ · min (95% CI, 0.0005-0.0044). Individuals undergoing masculinizing hormone therapy showed no significant changes in insulin sensitivity (Table 2, Fig. 3).

When considering feminizing and masculinizing hormone therapy together as 1 group, significant correlations (Spearman's rank correlation) were observed between Δ serum testosterone and Δ BMI (rho = 0.41; *P* < .05), lean body mass (rho = 0.52; *P* < .01), M-value [rho = −0.38; *P* < .05), and M/I ratio (rho = −0.51; *P* < .01; Fig. S1 (20)]. Moreover, Δ serum estradiol was correlated with Δ fat mass (rho = 0.46; *P* < .05) and lean body mass [rho = −0.49; *P* < .01; Fig. S1 (20)]. When stratified by feminizing and masculinizing hormone therapy, Δ serum testosterone correlated with Δ M/I ratio during feminizing hormone therapy (rho = −0.51, *P* < .05), whereas Δ serum estradiol correlated with Δ fat mass during masculinizing hormone therapy [rho = 0.58, *P* < .05; Table S4 (20)].

Significant correlations (Spearman's rank correlation) were also observed between Δ hematocrit [Table S5 (20)] and Δ M-value (rho = −0.44; *P* < .05) and M/I ratio (rho = −0.52; *P* < .01), considering feminizing and masculinizing hormone therapy as 1 group.

### Plasma Proteomics

As previously reported (19), feminizing hormone therapy was associated with 49 differentially expressed proteins (DEPs), while masculinizing hormone therapy was associated with 356 DEPs [Fig. S2 (20)]. Here we focused on the DEPs correlated with changes in insulin sensitivity.

#### Individual proteins associated with changes in M-value

**Figure 4.**
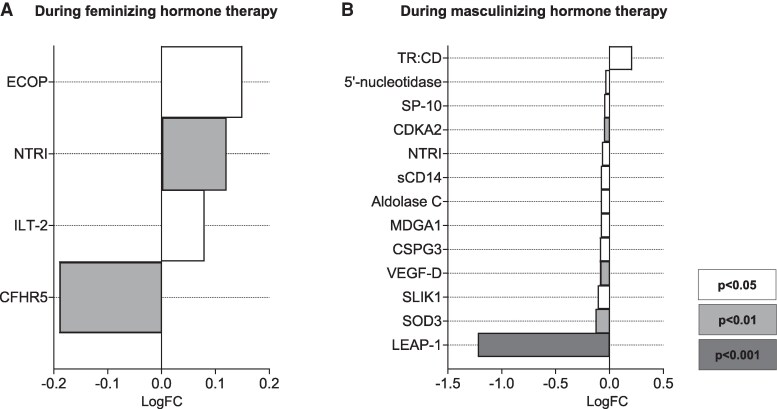
Log fold changes of the identified DEPs associated with changes in M-value during feminizing and masculinizing hormone therapy. The displayed proteins represent DEPs observed during feminizing (A) and masculinizing (B) hormone therapy, whose changes during sex hormone therapy (combining both feminizing and masculinizing therapies) were correlated with Δ M-value (Table 2). A Venn diagram summarizing the identification of these proteins can be found in Fig. S4 (20). * *P* < .05, **P < .01, ***P < .001. Abbreviations: CDKA2, cyclin-dependent kinase 2 associated protein 2; CFHR5, complement factor H-related protein 5; CSPG3, neurocan core protein; DEPs, differentially expressed proteins; ECOP, vesicular, overexpressed in cancer, prosurvival protein 1; ILT-2, leukocyte immunoglobulin-like receptor subfamily B member 1; LEAP-1, hepcidin; MDGA1, MAM domain-containing glycosylphosphatidylinositol anchor 1; NTRI, neurotrimin; sCD14, monocyte differentiation antigen CD14, soluble; SLIK1, SLIT and NTRK-like protein 1; SOD3, superoxide dismutase 3; SP-10, sperm protein 10; TR:CD, transferrin receptor protein 1:cytoplasmic domain; VEGF-D, vascular endothelial growth factor D.

In the full cohort (combining individuals undergoing both feminizing and masculinizing therapies), changes in M-value were correlated with 601 proteins, with the top 10 presented in Fig. S3 (20). When the analysis was restricted to DEPs associated specifically with either feminizing or masculinizing hormone therapy, 16 DEPs remained [Table S6 (20); Fig. 4)]. A Venn diagram summarizing the identification of these proteins can be found in Fig. S4 (20). Among the top 10 DEPs most significantly associated with Δ M-value, vascular endothelial growth factor D (VEGF-D), 5′-nucleotidase, SLIT and NTRK-like protein 1, leukocyte immunoglobulin-like receptor subfamily B member 1 (ILT-2), extracellular superoxide dismutase 3 (SOD3), neurocan core protein, sperm protein 10, MAM domain-containing glycosylphosphatidylinositol anchor protein 1, and hepcidin were positively associated with Δ M-value, while transferrin receptor protein 1:cytoplasmic domain was negatively associated with Δ M-value [Fig. 4; Table S6 (20)].

#### Ingenuity pathway analysis to identify pathways of interest

Of the previously identified differentially expressed pathways from the Ingenuity Pathway Analysis conducted using the SOMAScan assay protein set as the reference [61 for feminizing hormone therapy and 117 for masculinizing hormone therapy (19)], we focused here on those correlated with Δ M-value.

In the full cohort, combining individuals undergoing both feminizing and masculinizing hormone therapy, changes in M-value were associated with the upregulation or downregulation of 134 pathways. When the analysis was limited to differentially expressed pathways specific to either feminizing or masculinizing hormone therapy, 22 pathways were found to correlate with changes in M-value [Table S7 (20)]. A Venn diagram summarizing the identification of these pathways is available in Fig. S5 (20). The 2 pathways that were positively correlated with Δ M-value were regulation of eukaryotic initiation factor 4 and p70 ribosomal S6 kinase signaling and integrin cell surface interactions. The pathways that were negatively correlated with M-value were mainly involved in protein synthesis and translation [eukaryotic translation initiation, EIF2 signaling, response of EIF2AK4 (GCN2) to amino acid deficiency, eukaryotic translation elongation, eukaryotic translation termination, nonsense-mediated decay, and SRP-dependent cotranslational protein targeting to membrane], cell growth (mTOR signaling and p70 ribosomal S6 kinase signaling), selenoamino acid metabolism, cell proliferation, survival and differentiation (ERBB2 signaling, vascular endothelial growth factor signaling), cell response to hypoxia (HIF1α signaling), estrogen receptor signaling, eNOS signaling, immunoregulation (neutrophil degranulation), cancer signaling (colorectal cancer metastasis signaling), and, paradoxically, translocation of SLC2A4 (GLUT4) to the plasma membrane.

For all proteomic data, see Excel files A to H (20).

## Discussion

As early as 1980, an association between sex hormones and insulin sensitivity was demonstrated (22), and since then multiple studies have confirmed their important regulatory role (3-9). Our study adds to this evidence by directly assessing whole-body insulin sensitivity before and during sex hormone therapy in transgender individuals, using the hyperinsulinemic–euglycemic clamp and by exploring potential underlying mechanisms through plasma proteomics. We found that feminizing hormone therapy (estradiol with a GnRH analogue) improved whole-body insulin sensitivity, whereas masculinizing therapy with testosterone had no significant effect. These results align with the known protective role of estradiol in diseases such as type 2 diabetes, cardiovascular disease, and chronic kidney disease (2, 3).

Unlike earlier studies in transgender individuals, which often relied on surrogate markers such as HOMA-IR (10-12), our study used the gold-standard hyperinsulinemic-euglycemic clamp to directly assess insulin sensitivity. Furthermore, feminizing hormone therapy in our study included a GnRH analogue, instead of CPA, which has been shown to independently decrease insulin sensitivity (10-12, 23). By omitting CPA from the regimen, our study was able to reveal a beneficial effect of feminizing hormone therapy (estradiol with a GnRH analogue) on whole-body insulin sensitivity.

In line with most prior studies, we observed no significant change in insulin sensitivity following masculinizing therapy (10-12). However, a negative correlation between serum testosterone concentrations and insulin sensitivity suggests a negative effect of testosterone on insulin sensitivity that may not have been fully captured, possibly due to the short treatment duration, small sample size, or use of transdermal rather than intramuscular testosterone administration. Additionally, the absence of a marked decline in estradiol concentrations during masculinizing hormone therapy may have partially masked a clear testosterone effect.

Two cohort studies have assessed type 2 diabetes risk in transgender individuals. In the STRONG study, transgender women exhibited a higher risk compared with cisgender women; however, this difference was not observed among those receiving feminizing hormone therapy (24). Minority stress, the chronic stress experienced by marginalized groups, may contribute to this elevated risk of diabetes in transgender women compared with cisgender women, as it is also thought to play a role in the higher risk of cardiovascular disease transgender women face (25). The American College of Obstetricians and Gynecologists study reported no increased risk in transgender women receiving feminizing hormone therapy compared with their sex assigned at birth (cisgender men), while transgender men receiving masculinizing hormone therapy demonstrated a trend toward higher risk compared with cisgender women (26). These findings support the notion that feminizing hormone therapy may mitigate external risk factors, such as minority stress, and, potentially, restore the protective metabolic profile typically associated with estradiol.

To elucidate potentially involved mechanisms, we performed plasma proteomics and identified key proteins associated with changes in insulin sensitivity. Several changes in immunoregulatory proteins correlated positively with changes in insulin sensitivity, including ILT-2, which increased during feminizing hormone therapy, and VEGF-D, 5′-nucleotidase, and sCD14, which decreased during masculinizing hormone therapy. The changes in these proteins during sex hormone therapy seem largely sex hormone-driven, as they negatively correlated with testosterone and positively correlated with estradiol.

ILT-2, an inhibitory receptor on T-cells, may enhance insulin sensitivity by suppressing excessive immune activation and inflammation (27, 28). VEGF-D, a vascular endothelial growth factor, has previously also been associated with improved insulin sensitivity in individuals with obesity (29), potentially through its role in angiogenesis and reduction of macrophage-driven inflammation in adipose tissue (30). 5′-Nucleotidase catalyzes the conversion of AMP to adenosine (31), which has insulin-sensitizing effects by attenuating oxidative stress responses (32). Lastly, sCD14, a key regulator of immune response and inflammation, has been associated with improved insulin sensitivity in both human and animal studies (33, 34). sCD14 antagonizes CD14 receptor signaling, and by blocking cellular CD14 in macrophages and adipocytes, it reduces inflammatory pathways, including interleukin-1α and interleukin-1β, specifically in adipose tissue, leading to improved whole-body insulin action (33, 34). Collectively, these findings suggest that sex hormones may impact insulin sensitivity through an effect on inflammation and immunoregulation.

Iron homeostasis emerged as another potential contributor to sex hormone-associated changes in insulin sensitivity. Transferrin receptor protein 1:cytoplasmic domain, the cytoplasmic domain of transferrin receptor 1, a receptor essential for cellular iron uptake, increased during masculinizing hormone therapy and was inversely associated with insulin sensitivity. Hepcidin, a key regulator of iron homeostasis that inhibits iron absorption, decreased during masculinizing hormone therapy and was positively associated with insulin sensitivity. These changes during masculinizing hormone therapy, along with their strong correlation with serum testosterone, likely reflect testosterone-driven erythropoiesis. In line with this, hematocrit significantly increased during masculinizing and decreased during feminizing hormone therapy and was negatively correlated with insulin sensitivity, consistent with previous studies showing that increased hematocrit and erythropoiesis are associated with reduced insulin sensitivity (35-37). A proposed mechanism is that elevated hematocrit increases blood viscosity, thereby reducing capillary perfusion and limiting glucose delivery to the skeletal muscle interstitium (35). Overall, these findings indicate that iron homeostasis and testosterone-driven hematocrit changes may, in part, mediate sex hormones’ effects on insulin sensitivity.

Additionally, we observed a decrease in SOD3, an enzyme reducing oxidative stress, during masculinizing hormone therapy. SOD3 was positively correlated with estradiol, negatively correlated with testosterone, and positively associated with insulin sensitivity in our study. Additionally, SOD3 has shown protective effects against insulin resistance in rodent models (38). These findings suggest that its role in reducing oxidative stress may contribute to the sex hormone-associated changes in insulin sensitivity (38).

This study has several strengths, including the use of the hyperinsulinemic-euglycemic clamp, the gold standard for assessing insulin sensitivity, and proteomic analyses to explore underlying mechanisms. By leveraging a unique cohort of transgender individuals undergoing hormone therapy, it offers valuable insight into the metabolic effects of sex hormones. However, some limitations should be noted. First, the relatively small sample size limits statistical power and generalizability. Second, the 3-month follow-up period captured only early metabolic changes and does not inform long-term effects. Additionally, fixed hormone dosages as used in our protocol limited the assessment of dose-dependent effects. Furthermore, while GnRH analogues are not expected to directly affect insulin sensitivity (39), their use in feminizing hormone therapy may have influenced some observed effects. In addition, nearly all participants received transdermal sex hormones, meaning our findings are primarily applicable to this route of administration. However, in the context of feminizing hormone therapy, oral estradiol, due to hepatic first-pass effects, has been shown to improve insulin sensitivity even more than transdermal estradiol (4, 5), suggesting that our findings may also apply to oral estradiol. Moreover, insulin sensitivity was assessed only at the Amsterdam UMC site for logistical reasons. However, baseline serum sex hormone concentrations and body composition were comparable between individuals with and without insulin sensitivity measurements (data not shown), making selection bias unlikely. Finally, because the metabolic effects of estradiol and testosterone may be sex-specific (40), extrapolation of our findings from masculinizing and feminizing hormone therapy in individuals assigned female and male at birth, respectively, to the effects of sex hormones in cisgender individuals should be made with caution.

In conclusion, feminizing hormone therapy (estradiol with a GnRH analogue) improved insulin sensitivity, while masculinizing therapy did not show significant effects within the study window. Proteomic analyses pointed to roles for immunoregulation, inflammation, iron homeostasis, and oxidative stress in mediating sex hormone-driven insulin sensitivity changes. These findings emphasize the importance of sex hormones in metabolic health and support a precision medicine approach that considers sex hormonal context in managing insulin resistance in both cisgender and transgender individuals.

## Data Availability

The datasets generated and analyzed in the current study are available from the corresponding author upon reasonable request.
